# Impact of the COVID-19 pandemic on Swedish adolescents’ mental well-being: the role of impulsivity, sleep, spirituality, and self-esteem

**DOI:** 10.1186/s40359-025-03737-2

**Published:** 2025-12-02

**Authors:** Amir Pakpour, Marit Eriksson, Daniel Kwasi Ahorsu, Anders Broström, Staffan Bengtsson, Malin Jakobsson, Karina Huus

**Affiliations:** 1 Department of Nursing, School of Health and Welfare, Jönköping University, Gjuterigatan 5, 553 18 Jönköping, Jönköping, Sweden; 2 Futurum- the Academy for Health and Care, Region Jönköping County, Jönköping, Sweden; 3 Department of Health, Medicine and Caring Sciences, Linköping University, Linköping, Sweden; 4 Department of Special Education and Counseling, The Education University of Hong Kong, Hong Kong, China; 5 Department of Clinical Neurophysiology, Linköping University Hospital, Linköping, Sweden; 6 Department of Health and Caring Sciences, Western Norway University of Applied Sciences, Bergen, Vestlandet Norway; 7 CHILD-research group, Department of Nursing Science, School of Health and Welfare, Jönköping University, Jönköping, Sweden; 8 Department of Social Work, School of Health and Welfare, Jönköping University, Jönköping, Sweden

**Keywords:** Impulsivity, Sleep problems, Spiritual health, Self-esteem, COVID-19 impact, Mental well-being, Adolescents

## Abstract

**Background:**

This study examined the impact of the COVID-19 pandemic on mental well-being and the mediating roles of impulsivity, sleep problems, spiritual health, and self-esteem in this association.

**Methods:**

Swedish adolescents (*n* = 5548; boys = 50.9%) responded to an online survey on the impact of the COVID-19 pandemic, impulsivity, sleep problems, spiritual health, self-esteem, and mental well-being between September and October 2020.

**Results:**

The multigroup structural equation modeling (SEM) results for the whole group revealed a significant direct effect of COVID-19 pandemic on mental well-being and significant mediating effects of impulsivity, sleep problems, spiritual health, and self-esteem (*p* < 0.01). The mediators among girls (p values < 0.05) were similar to those among the whole group, but among boys, self-esteem (*p* = 0.186) was not significant.

**Conclusions:**

Generally, there are multiple pathways through which COVID-19 pandemic affects the well-being of adolescents. Therefore, there may be a need for psychoeducation and/or counseling on different coping strategies during infectious pandemics with a high risk of mortality to enhance mental well-being.

**Supplementary Information:**

The online version contains supplementary material available at 10.1186/s40359-025-03737-2.

## Introduction

Coronavirus 2019 (COVID-19) has had a global impact on various aspects of life, including health [1], economic activities [2, 3], and sociocultural lifestyles [4]. Due to the virulent nature of COVID-19, different countries instituted different measures such as lockdowns, physical distancing, and quarantines to break the transmission chain. Unfortunately, these preventive measures also had detrimental effects on the mental health of people [5], especially among adolescents [6–8]. Among adolescents, stress, social isolation and loneliness from preventive measures (e.g., lockdowns and quarantines) increased their risk of depression, anxiety, and sleep problems [6–8]. Furthermore, schools and other social support systems that served important coping, social and life functions for adolescents were closed because of either quarantine or lockdown which further worsened adolescents’ mental health [9, 10]. That is, lockdown prevents one from moving across a specified boundary, limiting socialization and other important activities, while quarantining further restrains one in confinement, usually a room, with very limited face-to-face contact, physical activity and other important life activities. These preventive measures reportedly had the most negative impact on people’s well-being [6–10], especially if compared to physical distancing. Physical distancing, approximately 1.5 m of space around all individuals to cut virus transmission, allows for most normal functioning but limits the number of people in a space at a given time.

The COVID-19 preventive measures implemented in Sweden are quite different from other countries. Compared to Sweden [11–13], most countries (e.g., Australia, China, USA) implemented stringent COVID-19 pandemic preventive measures such as quarantines and lockdowns, which had comparatively different impacts on citizens [14–17]. Previous studies from countries that implemented quarantines and lockdowns reported a direct COVID-19 pandemic impact on sleep quality among adolescents [14–17], although this finding can be complicated. Self-esteem was also affected by COVID-19 pandemic, although it was usually associated with other mental health conditions such as problematic social media use [18, 19]. Moreover, impulsivity increased among adolescents who were under social isolation during the pandemic [20, 21] or who had higher beliefs in COVID-19 conspiracy theories [22]. Spirituality (i.e., health-related quality of life concerning personal, spiritual, and religious beliefs) is mostly used as a coping mechanism; hence, it has been used to mitigate psychological distress during the COVID-19 pandemic [23, 24], thereby enhancing mental well-being [24, 25]. In addition, adolescent males had better mental well-being than females [26, 27]. Each of these studies provided insights into how impulsivity, sleep problems, poor spiritual health, and/or poor self-esteem were associated with either the impact of the COVID-19 pandemic or mental well-being. However, the segmented nature of their findings limits the comprehensive understanding of how all variables interact within a broader, integrated framework.

The transactional model of stress and coping [28] suggests that as an individual transacts with stress by continually appraising it, that individual uses appropriate coping strategies to manage the stressors perceived to exceed their resources [29, 30]. Also, the diathesis-stress model asserts that stress can trigger an underlying illness, especially in the absence of effective coping strategies [31]. COVID-19 has led to different stress levels [32, 33], and for adolescents, peer relationships at school and physical activities were important supportive and/or coping strategies [34] that helped their mental well-being. However, as Sweden implemented different preventive measures that took into account various population parameters, it is expected that adolescents will encounter minimal to none of these mental health challenges. For instance, there was no formal lockdown, although there was a recommendation for physical distancing and other ways to address COVID-19 at the individual level, such as good hand hygiene. Among adolescents, schools were still open with mandatory attendance for students in lower secondary school (grade 9 or below or 16 years and below), but those in upper secondary school and university switched to online schools [11–13].

Despite these unique preventive measures in Sweden, mental health challenges have been reported among adolescents, and even among children who were still attending school. A study among Swedish adolescents reported a relatively low overall impact of COVID-19 with notable sex differences. That is, Swedish adolescent males experienced elevated levels of anxiety, depression, sleep disturbances, anger, and increased illicit drug use compared to their female counterparts as the consequences of COVID-19 [35]. On the other hand, the females demonstrated an increase in several salutogenic behaviors than their male counterparts [35]. In other studies, stress, worries, and psychosomatic symptoms were reported among Swedish adolescents, and even among children [11–13, 32, 36, 37], with females having more worries, psychosomatic symptoms, poorer sleep, more anxiety, depression, anger, getting in more arguments, feeling lonelier, stressed, and less satisfied compared to males [11, 36, 37]. All these factors contributed to poorer mental well-being [11–13, 32, 36, 37], although inconsistencies exist between males and females [35, 37]. Reportedly, illicit drugs were used as coping strategies during COVID-19 [38, 39], with more males using them as compared to females [35, 38, 39]. This indicates that the nation, community, family, and individual had a role in intervening in the full effect of the COVID-19 pandemic on people’s mental well-being. Therefore, examining intervening variables such as impulsivity, sleep problems, spiritual health, and self-esteem may help with understanding the pathways through which the COVID-19 pandemic has affected the mental well-being of Swedish adolescents. These outcomes would help deal with future infectious diseases.

**Fig. 1:**
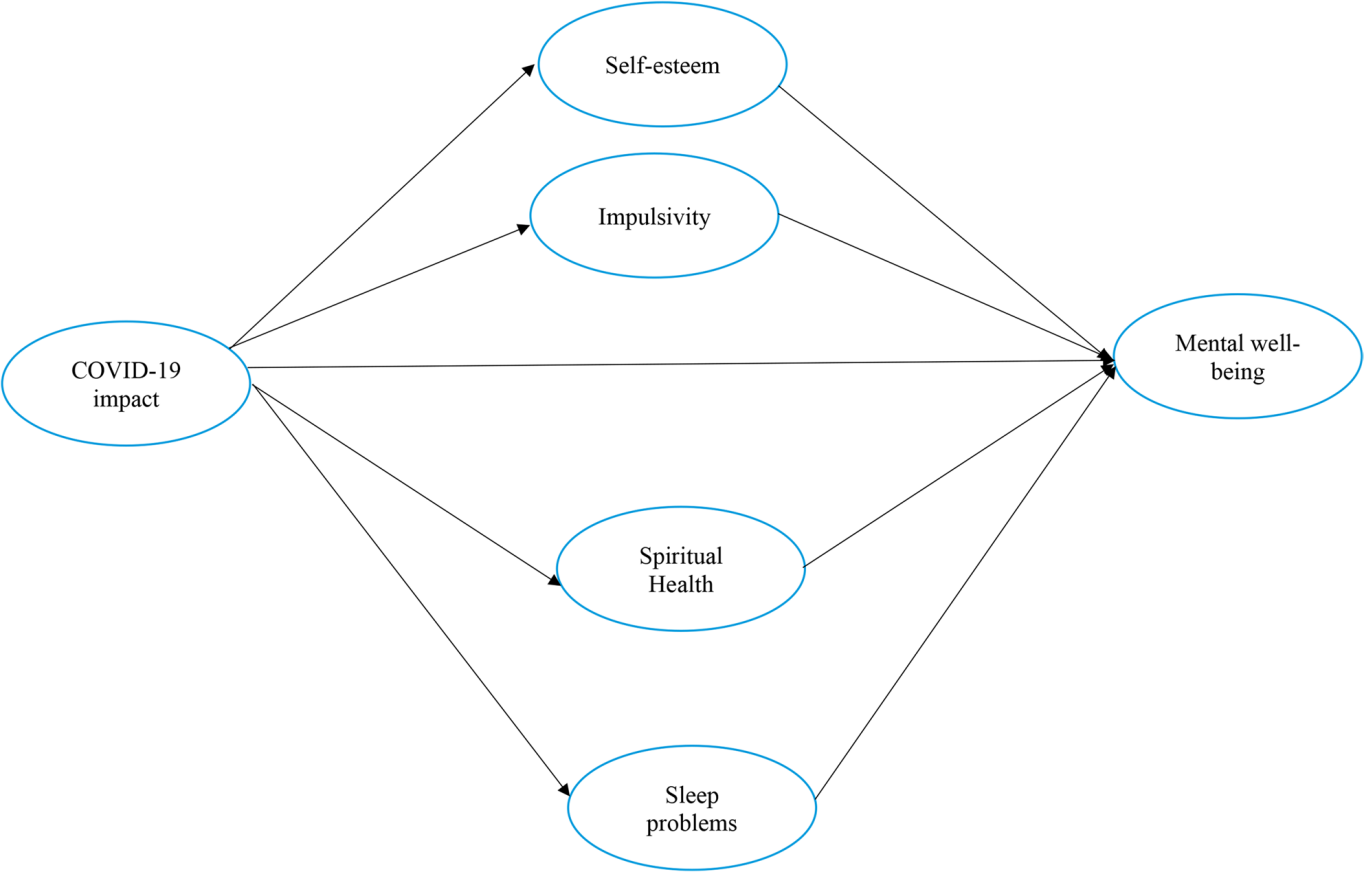
Hypothetical Model of COVID-19 Impact on Mental Well-being Among Swedish Adolescents, with Four Mediators: Self-esteem, Impulsivity, Spiritual Health, and Sleep Problems

As no known studies have examined the explicit mediating roles of impulsivity, sleep problems, spiritual health, and self-esteem in the association between the COVID-19 pandemic impact and mental well-being, the objectives of the present study are to examine (i) the relationships between the COVID-19 pandemic impact, impulsivity, sleep problems, spiritual health, self-esteem and mental well-being; (ii) the mediating roles of impulsivity, sleep problems, spiritual health, and self-esteem in the association between the COVID-19 pandemic impact and mental well-being; and (iii) whether the final accepted model remains invariant across sex groups (i.e., boys and girls which may be used interchangeably with males and females respectively) on mental well-being (Fig. 1).

## Methods

### Participants and procedure

This study used a cross-sectional survey design. The study was conducted between September and October 2020 on 5548 adolescents aged between 15 and 18 years in Jönköping County, Sweden. The data was collected among adolescents during school class times using an online platform (i.e., a web-based approach). Informed consents were obtained from all adolescents before the start of the study in order to conform with the Swedish Code of Statutes (2003:460, § 18). This Code of Statutes stipulates that participants between the ages of 15 and 18 years may consent and be included in a research without parental consent if they can independently understand what that particular research entails. The Swedish Ethical Review Authority (Dnr 2020–03173) approved the conduction of this study and has been reported elsewhere [40].

### Study measures

#### Short version of the Warwick-Edinburgh mental Well-being scale

This seven-item self-report scale is used to assess the mental well-being of individuals [41]. The items are ““I’ve been feeling good about myself.“, “I’ve been feeling optimistic about the future.” “I’ve been feeling cheerful.” “I’ve been interested in new things.” “I’ve been feeling loved.” “I’ve been able to make up my own mind about things.” and “I’ve been feeling calm and relaxed.” These items are rated on a five-point Likert scale ranging from 1 “None of the time” to 5 “All of the time”. All the item responses are summed together and then transformed into metric scores to obtain a total score [41, 42]. Therefore, higher scores indicate greater levels of mental well-being. This self-report scale has excellent psychometric properties (α =.900 and ω =.901) among adolescents in Sweden [40].

#### COVID-19 pandemic impact

This is an eight-item ad hoc measure for assessing the impact of the COVID-19 pandemic. The items are rated on a five-point Likert scale format ranging from 1 “much better than before” to 5 “much worse than before”. The main question of this measure was “How has the COVID-19 pandemic affected you in terms of?” The specific items were “Studies, friends, parents, siblings, relatives, leisure, physical health, and mental health”. All the item responses are summed to obtain a total response score, with higher scores indicating a worse impact of the COVID-19 pandemic. This measure had good internal consistency reliability (α = 0.849) for this study [40].

#### Sleep problems

A two-item scale was used to assess sleep problems among the adolescents. The items were “How often in the past 6 months have you had difficulty falling asleep?”, which was rated on a five-point Likert scale (rarely or never = 1, pretty much every day = 5), and “How do you think you sleep?”, which was rated on a five-point Likert scale format (very poor = 5, great = 1). All the response items are summed to obtain a total response score, with higher scores indicating greater sleep problems. This measure has acceptable internal consistency reliability (α = 0.774) for this study.

#### Brief measure of spiritual health

Spiritual health can be considered an essential part of a person’s well-being. Its definition can vary depending on cultural and philosophical contexts, but it is a multidimensional concept that encompasses dimensions like transcendence, meaningfulness, and purposefulness. In the current study, individuals’ sense-making was focused on measuring spiritual health. For that reason, a four-item brief measure was used to assess adolescents’ spiritual health. These items are reflective of the World Health Organization Quality of Life Spiritual Religious and Personal Belief (WHOQOL-SRPB) scale [43]. The items include “I feel that my life is meaningful”, “I feel that my life has a purpose”, “I have a belief or conviction that gives me comfort/relief in everyday life” and “I feel a sense of hope about my life”. The items of this scale are rated on a five-point Likert scale ranging from completely disagree (1) to completely agree (5). All the item responses are summed to obtain a total response score, with higher scores indicating greater spiritual health. In this study, the measure has good internal consistency reliability (α = 0.827).

#### Impulsivity

A three-item scale was used to assess impulsivity among the adolescents. The items were “I am impulsive and act without thinking”, “I become very angry and am often in a bad mood”, and “I fight or argue a lot. I can force others to do what I want”. The items are rated on a three-point Likert scale ranging from disagree (1) to agree (3). All the item responses are summed to obtain a total response score, with higher scores indicating greater impulsivity. In this study, the measure had almost acceptable internal consistency reliability (α = 0.636).

#### Self-esteem

A three-item scale was used to assess the self-esteem of the participants. The items were “I like myself”, “I am good enough as I am”, and “Other people of my age like me”. The items are rated on a five-point Likert scale ranging from completely disagree (1) to completely agree (5). All the item responses are summed to obtain a total response score, with higher scores indicating higher self-esteem. A previous study reported good internal consistency (α = 0.89) among adolescents in Nordic countries, including Swedish adolescents [21]. In this study, the measure had good internal consistency reliability (α = 0.846).

### Data analysis

The descriptive statistics (e.g., mean and standard deviation) were used to present the characteristics of the participants. Mann‒Whitney U tests were used to test differences in study measures across sex groups. Moreover, p-values from multiple comparisons were corrected using the Benjamini–Hochberg method for false discovery rate. Spearman’s rank-order correlation analysis was conducted to investigate the relationships between the study variables. The internal consistency of the study measures was assessed using Cronbach’s alpha and McDonald’s omega, with values equal to or greater than 0.70 deemed acceptable. The data were analyzed using the recommended two-step statistical procedure [44]. The theoretical framework of each study measure was first examined using confirmatory factor analysis (CFA) with the diagonally weighted least square estimator. Afterwards, structural equation modeling (SEM) with full information maximum likelihood was used to investigate direct and indirect associations between the impacts of the COVID-19 pandemic and mental well-being among Swedish adolescents. Model fit was assessed using a comparative fit index (CFI) with an index greater than 0.95, Tucker‒Lewis index (TLI) with an index greater than 0.95, root mean square error of approximation (RMSEA) with an index less than 0.06, and standardized root mean square residual (SRMR) with an index less than 0.08. The indirect associations between the impacts of the COVID-19 pandemic and mental well-being were estimated based on 95% bias-corrected bootstrap confidence intervals (CIs) with 5,000 bootstrap resamples. Additionally, multigroup SEM was performed to assess the invariance of the final hypothesized model across sex subgroups. Model invariance across sex was evaluated using ΔCFI > −0.02, ΔRMSEA < 0.02, and ΔSRMR < 0.03 [45]. The statistical analyses were conducted using SPSS version 27, AMOS version 24 and JASP version 0.18.3.0.

## Results

The descriptive statistics in Table 1 show the measures used in this study. Furthermore, the reliability (internal consistency) coefficients were acceptable, especially for the Cronbach’s alpha coefficients.

Sex comparisons across different study measures are shown in Table 1. According to the Mann‒Whitney U test statistics and corresponding Z scores, compared with boys, girls reported significantly greater levels of impulsivity (mean ± SD: 4.46 ± 1.38 for girls vs. 4.05 ± 1.28 for boys, *p* < 0.001), sleep problems (4.75 ± 2.11 for girls vs. 4.32 ± 2.14 for boys, *p* < 0.001), and the impact of the COVID‒19 pandemic (26.26 **±** 4.24 for girls vs. 25.29 **±** 5.10 for boys, *p* < 0.001). Furthermore, compared with boys, girls reported lower levels of spiritual health (mean ± SD: 15.09 ± 4.0 for girls vs. 16.65 ± 3.65 for boys, *p* < 0.001), self-esteem (mean ± SD: 11.45 ± 2.50 for girls vs. 12.75 ± 2.27 for boys, *p* < 0.001), and mental well-being (mean ± SD: 23.80 ± 4.84 for girls vs. 27.24 ± 5.18 for boys, *p* < 0.001). The correlations between the study variables shown in Table 2 revealed that there were small to large significant relationships between the study variables (p values < 0.01). The impact of the COVID-19 pandemic was positively related to impulsivity (*r* = 0.0114) and sleep problems (*r* = 0.137) but negatively related to spiritual health (*r*=−0.129), self-esteem (*r*=−0.157) and mental well-being (*r*=−0.198). Sleep problems were positively related to impulsivity (*r* = 0.288) but negatively related to spiritual health (*r*=−0.241), self-esteem (*r*=−0.278) and mental well-being (*r*=−0.370). Sleep problems were negatively related to spiritual health (*r*=−0.312), self-esteem (*r*=−0.361) and mental well-being (*r*=−0.443). Finally, there were positive relationships between spiritual health, self-esteem and mental well-being (*r* = 0.581–0.723).

**Table 1:** Descriptive statistics of the study measures

	Range	Mean	SD	α	ω	Boys Mean ± SD	Girls Mean ± SD	Z (*P* value) for sex comparison^a^
1. COVID-19 impact	8–40	25.78	4.74	0.849	0.846	25.29 **±** 5.10	26.26 **±** 4.24	−10.62 (< 0.001)
2. Impulsivity	3–9	4.26	1.35	0.636	0.660	4.05 ± 1.28	4.46 ± 1.38	−12.31(< 0.001)
3. Sleep problems	2–10	4.54	2.14	0.774	-	4.32 ± 2.14	4.75 ± 2.11	−7.63(< 0.001)
4. Spiritual health	4–20	15.84	3.93	0.827	0.828	16.65 ± 3.65	15.09 ± 4.0	−15.17(< 0.001)
5. Self-esteem	3–15	12.146	2.60	0.846	0.871	12.75 ± 2.27	11.45 ± 2.50	−22.13(< 0.001)
6. Mental well-being	7–35	25.524	5.32	0.900	0.901	27.24 ± 5.18	23.80 ± 4.84	−23.48(< 0.001)

^**a**^ based on the Mann-Whitney U test statistics

Note: α=Cronbach’s alpha coefficient, ω=McDonald’s omega, sex refers to boys and girls. All p values were corrected by the Benjamini–Hochberg False Discovery Rate

**Table 2:** The spearman rank correlation of study variables

	1	2	3	4	5	6
1. COVID-19 impact	1	0.114	0.137	−0.129	−0.157	−0.198
2. Impulsivity	-	1	0.288	−0.241	−0.278	−0.370
3. Sleep problems	-	-	1	−0.312	−0.361	−0.443
4. Spiritual health	-	-	-	1	0.581	0.588
5. Self-esteem	-	-	-	-	1	0.723
6. Mental well-being	-	-	-	-	-	1

All p values are significant at *p* < 0.01

The CFA results shown in Table 3 revealed that there were acceptable fit indices for the COVID-19 pandemic impact measure (CFI = 0.968, TLI = 0.956, RMSEA = 0.056, and SRMR = 0.077), impulsivity measure (CFI = 1, TLI = 1, RMSEA = 0, SRMR = 0), spiritual health measure (CFI = 0.999, TLI = 0.998, RMSEA = 0.019, SRMR = 0.013), self-esteem measure (CFI = 1, TLI = 1, RMSEA = 0, SRMR = 0) and mental well-being measure (CFI = 0.998, TLI = 0.997, RMSEA = 0.027, SRMR = 0.027).

**Table 3:** Results of the confirmatory factor analysis among study measures

	Chi-square	df	CFI	TLI	RMSEA	SRMR
1. COVID-19 impact	316.18	20	0.968	0.956	0.056	0.077
2. Impulsivity	0	0	1	1	0	0
3. Sleep problems	NA	-	-	-	-	-
4. Spiritual health	5.77	2	0.999	0.998	0.019	0.013
5. Self-esteem	0	0	1	1	0	0
6. Mental well-being	66.22	14	0.998	0.997	0.027	0.027

*CFI* comparative fit index, *TLI* Tucker-Lewis index, *RMSEA* root mean square error of approximation,*SRMR* standardized root mean square residual

The SEM results shown in Table 4 revealed that for the whole group, there was a significant direct negative effect of the COVID-19 pandemic on mental well-being (unstandardized coefficient = −0.056, *p* < 0.001), a significant indirect negative effect (unstandardized coefficient = −0.195, *p* < 0.001), and a total effect (unstandardized coefficient = −0.251, *p* < 0.001) on mental well-being. Additionally, there was a significant positive direct effect of the COVID-19 pandemic on sleep problems (unstandardized coefficient = 0.308, *p* < 0.001) and impulsivity (unstandardized coefficient = 0.111, *p* < 0.001) but a negative direct effect on spiritual health (unstandardized coefficient = −0.077, *p* = 0.003) and self-esteem (unstandardized coefficient = −0.117, *p* < 0.001). Furthermore, sleep problems (unstandardized coefficient = −0.105, *p* < 0.001) and impulsivity (unstandardized coefficient = −0.198, *p* < 0.001) had significant negative direct effects on mental well-being, while spiritual health (unstandardized coefficient = 0.181, *p* < 0.001) and self-esteem (unstandardized coefficient = 0.296, *p* < 0.001) had significant direct positive effects on mental well-being. The squared multiple correlation (R^2^) for this model was 0.691 (i.e., 69.1% of the predicted variance was explained by this model).

**Table 4:** Direct, indirect, and total standard effects

Parameter	Total Effect (*p*-value)	Direct Effect (*p*-value)	Indirect Effect (*p*-value)	Bootstrapping SE (LLCI, ULCI)
Whole sample				
COVID-19 impact → mental well-being	−0.251 (*p* < 0.001)	−0.056 (*p* < 0.001)	−0.195 (*p* < 0.001)	0.020 (−0.235, −0.158)
COVID-19 impact → sleep problems	-	0.308 (*p* < 0.001)	-	-
COVID-19 impact → spiritual health	-	−0.077 (0.003)	-	-
COVID-19 impact → impulsivity	-	0.111 (*p* < 0.001)	-	-
COVID-19 impact → self-esteem	-	−0.117 (*p* < 0.001)	-	-
Sleep problems→ mental well-being	-	−0.105 (*p* < 0.001)	-	-
Spiritual Health→ mental well-being	-	0.181 (*p* < 0.001)	-	-
Impulsivity → mental well-being	-	−0.198 (*p* < 0.001)	-	-
Self-Esteem → mental well-being	-	0.296 (*p* < 0.001)	-	-
Boys				
COVID-19 impact → mental well-being	−0.130 (*p* < 0.001)	−0.037 (*p* < 0.001)	−0.093 (*p* < 0.001)	0.019 (−0.133, −0.057)
COVID-19 impact → sleep problems	-	0.204 (*p* < 0.001)	-	-
COVID-19 impact → spiritual health	-	−0.079 (0.020)	-	-
COVID-19 impact → impulsivity	-	0.059 (0.007)	-	-
COVID-19 impact → self-esteem	-	−0.044 (0.186)	-	-
Sleep problems→ mental well-being	-	−0.087 (*p* < 0.001)	-	-
Spiritual health → mental well-being	-	0.139 (*p* < 0.001)	-	-
Impulsivity → mental well-being	-	−0.149 (*p* < 0.001)	-	-
Self-esteem → mental well-being	-	0.329 (*p* < 0.001)	-	-
Girls				
COVID-19 impact → mental well-being	−0.289 (*p* < 0.001)	−0.056 (0.009)	−0.233 (*p* < 0.001)	0.030 (−0.295, −0.176)
COVID-19 impact → sleep problems	-	0.390 (*p* < 0.001)	-	-
COVID-19 impact → spiritual health	-	−0.072 (0.050)	-	-
COVID-19 impact → impulsivity	-	0.127 (*p* < 0.001)	-	-
COVID-19 impact → self-esteem	-	−0.128 (0.007)	-	-
Sleep problems→ mental well-being	-	−0.116 (*p* < 0.001)	-	-
Spiritual health→ mental well-being	-	0.228 (*p* < 0.001)	-	-
Impulsivity → mental well-being	-	−0.127 (*p* < 0.001)	-	-
Self-esteem → mental well-being	-	0.234 (*p* < 0.001)	-	-

Unstandardized values were shown in the table Squared Multiple Correlation for mental well-being for whole sample, 0.691, boys 0.664 and girls 0.662 Mediation analysis was conducted employing 5000 bootstrapping iterations, and percentile confidence intervals were computed using AMOS

For boys only, there was a significant negative direct effect of the COVID-19 pandemic on mental well-being (unstandardized coefficient = −0.037, *p* < 0.001), a negative indirect effect (unstandardized coefficient = −0.093, *p* < 0.001), and a significant negative total effect of all the models on mental well-being (unstandardized coefficient = −0.130, *p* < 0.001). Additionally, there were significant positive direct effects of the COVID-19 pandemic on sleep quality (unstandardized coefficient = 0.204, *p* < 0.001) and impulsivity (unstandardized coefficient = 0.059, *p* = 0.007) but a negative direct effect on spiritual health (unstandardized coefficient = −0.079, *p* = 0.020), while no significant effect was found on self-esteem (unstandardized coefficient = −0.044, *p* = 0.186). Furthermore, sleep problems (unstandardized coefficient = −0.087, *p* < 0.001) and impulsivity (unstandardized coefficient = −0.149, *p* < 0.001) had significant negative direct effects on mental well-being, while spiritual health (unstandardized coefficient = 0.139, *p* < 0.001) and self-esteem (unstandardized coefficient = 0.329, *p* < 0.001) had significant positive direct effects on mental well-being. The squared multiple correlation (R^2^) for this model was 0.664 (i.e., 66.4% of the predicted variance was explained by this model).

For girls only, there was a significant negative direct effect of the COVID-19 pandemic on mental well-being (unstandardized coefficient = −0.056, *p* = 0.009), a negative indirect effect (unstandardized coefficient = −0.233, *p* < 0.001), and a significant negative total effect of all the models on mental well-being (unstandardized coefficient = −0.289, *p* < 0.001). Additionally, there were significant positive direct effects of the COVID-19 pandemic on sleep quality (unstandardized coefficient = 0.390, *p* < 0.001) and impulsivity (unstandardized coefficient = 0.127, *p* < 0.001) but negative direct effects on spiritual health (unstandardized coefficient = −0.072, *p* = 0.050) and self-esteem (unstandardized coefficient = −0.128, *p* = 0.007). Furthermore, sleep problems (unstandardized coefficient = −0.116, *p* < 0.001) and impulsivity (unstandardized coefficient = −0.127, *p* < 0.001) had significant direct negative effects on mental well-being, but spiritual health (unstandardized coefficient = 0.228, *p* < 0.001) and self-esteem (unstandardized coefficient = 0.234, *p* < 0.001) had significant direct positive effects on mental well-being. The squared multiple correlation (R^2^) for this model was 0.662 (i.e., 66.2% of the predicted variance was explained by this model).

The multigroup SEM results on invariance evaluation across groups, shown in Table S1 (see Table S1 in the Appendix), revealed good fit indices. The unconstrained indices for sex groups (boy or girl) were χ^2^ (df) = 10004.89 (933), CFI = 0.940, TLI = 0.932, RMSEA = 0.030, and SRMR = 0.057. Changes across the models (i.e., measurement weights, measurement intercepts, structural weights, structural covariances, structural residuals and measurement residuals) were found to meet the cutoff values for ΔCFI > −0.02, ΔRMSEA < 0.02, and ΔSRMR < 0.03, indicating invariance across sex groups on mental well-being (See Appendix S1 for more information).

## Discussion

The present cross-sectional study examined the impact of the COVID-19 pandemic on Swedish adolescents’ mental well-being, taking into consideration that they only used physical distancing and online classes among school students compared to most countries that implemented lockdowns, quarantines, and physical distancing. Specifically, this study examined the mediating roles of impulsivity, sleep problems, spiritual health, and self-esteem in the association between the impact of the COVID-19 pandemic and mental well-being, as well as evaluation invariance across sex groups. The results revealed that there were significant relationships (i.e., positive to negative, small to large effects) between all the studied variables, indicating that all the studied variables are intricately related to each other.

Specifically, the impact of the COVID-19 pandemic was positively related to impulsivity and sleep problems but negatively related to spiritual health, self-esteem and mental well-being. These findings indicate that a worse impact of the COVID-19 pandemic most likely leads to greater impulsivity, greater sleep problems, poorer spiritual health, lower self-esteem, poorer mental well-being and/or vice versa. This suggests that stressful events such as COVID-19 can significantly influence other mental health conditions, especially when individuals use maladaptive coping strategies [28, 38, 39]. Additionally, greater impulsivity is related to more sleep problems but lower spiritual health, self-esteem and mental well-being, suggesting that greater impulsivity most likely leads to greater sleep problems, poorer spiritual health, lower self-esteem, and poorer mental well-being and/or vice versa. A previous study revealed that pleasant, direct and empathetic communication from parents was able to help, control and guide adolescents’ impulsivity levels and eventual mental health during the COVID-19 pandemic [20]. Furthermore, sleep problems were negatively related to spiritual health, self-esteem and mental well-being, indicating that greater sleep problems most likely lead to poorer spiritual health, lower self-esteem, and poorer mental well-being and/or vice versa. Additionally, there was a positive relationship between spiritual health, self-esteem and mental well-being, indicating that greater spiritual health most likely leads to higher self-esteem and greater mental well-being and/or vice versa. Overall, these relationships suggest the complexities and relatedness of these variables such that the emergence of one variable may influence another variable. This is understandable, as some adolescents expressed experiencing stress, worries, sleep problems and similar others even though they experienced a comparatively “relaxed” form of COVID-19 preventive measures [11–13, 32, 36, 37]. Importantly, these relationships are supported by previous studies that revealed significant relationships between COVID-19 pandemic impact, impulsivity, sleep problems, spiritual health, self-esteem and mental well-being, although there were different COVID-19 preventive measures [14–21].

Furthermore, the SEM revealed that the impact of the COVID-19 pandemic directly affected mental well-being such that a worse COVID-19 impact may directly lead to poorer mental well-being. Additionally, the results revealed that the impact of the COVID-19 pandemic directly affected sleep, spiritual health, impulsivity, and self-esteem, and these variables also directly affected mental well-being. These findings indicate an indirect impact of the COVID-19 pandemic on mental well-being via sleep, spiritual health, impulsivity, and self-esteem. This suggests that not only can the COVID-19 pandemic directly affect mental well-being but also indirectly affect mental well-being through other factors, such as sleep quality, spiritual health, impulsivity, and self-esteem. This further indicates that people with a worse impact of the COVID-19 pandemic have a greater chance of having poorer mental well-being, as there are at least four pathways (i.e., sleep problems, spiritual health, impulsivity, and self-esteem) through which the COVID-19 pandemic may affect mental well-being. There might be a lot of reasons for the perpetuating impact of COVID-19 on mental well-being via sleep problems, spiritual health, impulsivity, and self-esteem. However, one of the main reasons will be the use of ineffective or maladaptive coping strategies [28, 38, 39]. Spiritual health practices may enhance mental well-being but may not be enough to fully mitigate the impact of COVID-19. This is supported by the transactional model of stress and coping and the diathesis-stress model [28, 31]. The findings on mediation are novel, as there is no known study among adolescents and only one study among adults that examined an aspect (i.e., spirituality mediating the association between fear of COVID-19 and mental health) of these mediations [46].

In addition, a separate analysis of adolescent boys and girls revealed that both sexes had findings similar to those of the whole group, except that among boys, the impact of the COVID-19 pandemic did not directly affect their self-esteem, even though self-esteem directly affected mental well-being. This indicates that the impact of the COVID-19 pandemic did not affect boys’ mental well-being through their self-esteem. This suggests that COVID-19 affects Swedish adolescent boys differently from their female counterparts in that there are at least four pathways through which the COVID-19 pandemic may impact the mental well-being of female Swedish adolescents compared to that of Swedish adolescent boys (i.e., at least three pathways minus self-esteem). The greater number of pathways for mental well-being among girls partly explains why they experienced poorer mental well-being even during the COVID-19 pandemic, as supported by previous studies [11, 26, 27, 36, 37]. Furthermore, the girls had comparatively stronger indirect effects, which may also account for the number of pathways and their significant effects on their well-being. Nonetheless, further studies, including qualitative studies, may be needed to examine the reasons behind boys’ self-esteem during the COVID-19 pandemic compared to girls’ self-esteem. The Mann‒Whitney U test also revealed that girls had significantly greater impulsivity, sleep problems and COVID‒19 pandemic impacts but poorer spiritual health, self-esteem, and mental well-being than boys. These findings further explain the direct differences between sexes in terms of the strength of the associations between mental health variables and the number of pathways to mental well-being. These findings are in line with those of previous studies [16, 26, 27], although less is known about why sex differences exist.

The invariance evaluation revealed that there was no inconsistency in mental well-being as a construct across sex groups (boys and girls). This indicates that the mental well-being of Swedish adolescents is invariant based on sex. That is, the concept of mental well-being does not change depending on an individual’s sex (i.e., boy or girl), which further suggests that both boys and girls experience the same concept concerning mental well-being. This is a positive finding that further suggests that health experts and health communicators may have to address Swedish adolescents as a unit on issues about mental well-being. This will make mental health education simple and focused. This finding also suggested that adaptive coping strategies taught by health experts for mental well-being may be similar for both boys and girls, although there may be preferential differences between the sexes. However, there may be a need for future studies to explore other factors that may vary in mental well-being among Swedish adolescents to also address mental well-being appropriately. This recommendation is based on previous studies that reported significant differences between boys and girls in mental health [11, 26, 27, 36, 37] as well as the multiple pathway findings in this study.

### Limitations, strengths, and implications

The current study had several limitations. A cross-sectional survey design was adopted for the current study. Cross-sectional studies are known for their limitations in making causal inferences. Therefore, consumers of this study should take note of and be cautious in overextending the findings. Additionally, the participants used in this study were adolescents, so care must be taken in overgeneralizing the findings across the other age groups. Furthermore, some of the scales (e.g., impulsivity, sleep problems, and self-esteem) have not been validated. Therefore, future studies can extensively validate these scales. Despite these limitations, the current study has several strengths. First, the current study generated novel findings in terms of the mediating factors in the association between the impact of the COVID-19 pandemic and mental well-being. Specifically, there were at least four pathways through which the COVID-19 pandemic affects the mental well-being of adolescents. Moreover, there were differences in how the COVID-19 pandemic affected mental well-being between boys (i.e., at least three pathways) and girls (i.e., at least four pathways). This implies that female adolescents have a greater chance of having poorer mental well-being due to their four pathways than male adolescents with three pathways. Therefore, it is recommended that all adolescents be psycho-educated on different adaptive ways of coping with stress and challenges, including infectious pandemics such as COVID-19, so as to enhance their mental well-being during those challenging times.

## Conclusion

The present study examined the impact of the COVID-19 pandemic on Swedish adolescents’ mental well-being by specifically examining the mediating roles of impulsivity, sleep problems, spiritual health, and self-esteem in the association between the COVID-19 pandemic impact and mental well-being, as well as evaluation invariance across sex groups. Impulsivity, sleep problems, spiritual health, and self-esteem were found to serve as mediators of the association between the COVID-19 pandemic and mental well-being. Furthermore, the mediators were different between boys (i.e., impulsivity, sleep problems, and spiritual health) and girls (i.e., impulsivity, sleep problems, spiritual health, and self-esteem). Nonetheless, the mental well-being of Swedish adolescents is invariant based on sex. These findings suggest that there are multiple ways through which the COVID-19 pandemic may affect the well-being of adolescents in Sweden. Therefore, there may be a need for psychoeducation and/or counseling, especially on different ways of coping with infectious pandemics such as COVID-19, to enhance mental well-being.

## Supplementary Information

Supplementary Material 1.

## Data Availability

The datasets generated during and/or analysed during the current study are available from the corresponding author on reasonable request.

## Abbreviations

CFA: Confirmatory factor analysis
CFI: Comparative fit index
CI: Confidence intervals
COVID-19: Coronavirus disease 2019
RMSEA: Root mean square error of approximation
SD: Standard deviation
SEM: Structural equation modeling
SRMR: Standardized root mean square residual
TLI: Tucker‒lewis index

## References

[1] Fazeli S, Mohammadi Zeidi I, Lin CY, Namdar P, Griffiths MD, Ahorsu DK, et al. Depression, anxiety, and stress mediate the associations between internet gaming disorder, insomnia, and quality of life during the COVID-19 outbreak. Addict Behav Rep. 2020;12:100307. 10.1016/j.abrep.2020.100307. 33110934 10.1016/j.abrep.2020.100307 PMC7581367

[2] Asante LA, Mills RO. Exploring the socio-economic impact of COVID-19 pandemic in marketplaces in urban Ghana. Afr Spectr. 2020;55(2):170–81.

[3] Nicola M, Alsafi Z, Sohrabi C, Kerwan A, Al-Jabir A, Iosifidis C, Agha M, Agha R. The socio-economic implications of the coronavirus pandemic (COVID-19): a review. Int J Surg. 2020;78:185–93. 10.1016/j.ijsu.2020.04.018. 32305533 10.1016/j.ijsu.2020.04.018 PMC7162753

[4] Van Lancker W, Parolin Z. COVID-19, school closures, and child poverty: a social crisis in the making. Lancet Public Health. 2020;5(5):e243–4. 10.1016/S2468-2667(20)30084-0. 32275858 10.1016/S2468-2667(20)30084-0 PMC7141480

[5] Brooks SK, Webster RK, Smith LE, Woodland L, Wessely S, Greenberg N, Rubin GJ. The psychological impact of quarantine and how to reduce it: rapid review of the evidence. Lancet. 2020;395(10227):912–20. 10.1016/S0140-6736(20)30460-8. 32112714 10.1016/S0140-6736(20)30460-8 PMC7158942

[6] Loades ME, Chatburn E, Higson-Sweeney N, Reynolds S, Shafran R, Brigden A, Linney C, McManus MN, Borwick C, Crawley E. Rapid systematic review: the impact of social isolation and loneliness on the mental health of children and adolescents in the context of COVID-19. J Am Acad Child Adolesc Psychiatry. 2020;59(11):1218–e12393. 10.1016/j.jaac.2020.05.009. 32504808 10.1016/j.jaac.2020.05.009 PMC7267797

[7] Nearchou F, Flinn C, Niland R, Subramaniam SS, Hennessy E. Exploring the impact of COVID-19 on mental health outcomes in children and adolescents: a systematic review. Int J Environ Res Public Health. 2020;17(22):8479. 10.3390/ijerph17228479. 33207689 10.3390/ijerph17228479 PMC7698263

[8] Singh S, Roy D, Sinha K, Parveen S, Sharma G, Joshi G. Impact of COVID-19 and lockdown on mental health of children and adolescents: a narrative review with recommendations. Psychiatry Res. 2020;293:113429. 10.1016/j.psychres.2020.113429. 32882598 10.1016/j.psychres.2020.113429 PMC7444649

[9] Cowie H, Myers CA. The impact of the COVID-19 pandemic on the mental health and well-being of children and young people. Child Soc. 2021;35(1):62–74. 10.1111/chso.12430. 33362362 10.1111/chso.12430 PMC7753823

[10] Naff D, Williams S, Furman-Darby J, Yeung M. The mental health impacts of COVID-19 on PK–12 students: a systematic review of emerging literature. AERA Open. 2022;8:23328584221084722. 10.1177/23328584221084722.

[11] Hagquist C. Worry and psychosomatic problems among adolescents in Sweden in the wake of the covid-19 pandemic: unequal patterns among sociodemographic groups? J Adolesc Health. 2023;72(5):688–95. 10.1016/j.jadohealth.2022.12.013. 36781326 10.1016/j.jadohealth.2022.12.013 PMC9918865

[12] Sarkadi A, Sahlin Torp L, Pérez-Aronsson A, Warner G. Children’s expressions of worry during the COVID-19 pandemic in Sweden. J Pediatr Psychol. 2021;46(8):939–49. 10.1093/jpepsy/jsab060. 34383921 10.1093/jpepsy/jsab060 PMC8376257

[13] Tishelman C, Degen JL, Weiss Goitiandía S, Kleijberg M, Kleeberg-Niepage A. A qualitative serial analysis of drawings by thirteen-to fifteen-year-old adolescents in Sweden about the first wave of the COVID-19 pandemic. Qual Health Res. 2022;32(8–9):1370–85. 10.1177/10497323221101978. 35599585 10.1177/10497323221101978 PMC9350847

[14] Deng J, Zhou F, Hou W, Heybati K, Lohit S, Abbas U, Silver Z, Wong CY, Chang O, Huang E, Zuo QK, Moskalyk M, Ramaraju HB, Heybati S. Prevalence of mental health symptoms in children and adolescents during the COVID-19 pandemic: a meta-analysis. Ann N Y Acad Sci. 2023;1520(1):53–73. 10.1111/nyas.14947. 36537131 10.1111/nyas.14947 PMC9880764

[15] Jahrami HA, Alhaj OA, Humood AM, Alenezi AF, Fekih-Romdhane F, AlRasheed MM, et al. Sleep disturbances during the COVID-19 pandemic: a systematic review, meta-analysis, and meta-regression. Sleep Med Rev. 2022;62:101591. 10.1016/j.smrv.2022.101591. 35131664 10.1016/j.smrv.2022.101591 PMC8782754

[16] Rocha S, Fuligni A. The impact of the COVID-19 pandemic on adolescent sleep behavior. Curr Opin Psychol. 2023;52:101648. 10.1016/j.copsyc.2023.101648. 37454639 10.1016/j.copsyc.2023.101648 PMC10290178

[17] Sharma M, Aggarwal S, Madaan P, Saini L, Bhutani M. Impact of COVID-19 pandemic on sleep in children and adolescents: a systematic review and meta-analysis. Sleep Med. 2021;84:259–67. 10.1016/j.sleep.2021.06.002. 34182354 10.1016/j.sleep.2021.06.002 PMC8687656

[18] Ramsey N, Obeidallah M, Abraham A. Impact of COVID-19 on adolescent health and use of social media. Curr Opin Pediatr. 2023;35(3):362–67. 10.1097/MOP.0000000000001248. 37036294 10.1097/MOP.0000000000001248 PMC10155612

[19] Vall-Roqué H, Andrés A, Saldaña C. The impact of COVID-19 lockdown on social network sites use, body image disturbances and self-esteem among adolescent and young women. Prog Neuropsychopharmacol Biol Psychiatry. 2021;110:110293. 10.1016/j.pnpbp.2021.110293. 33662532 10.1016/j.pnpbp.2021.110293 PMC8569938

[20] García EE, Castro-Santisteban M, Torres DF, Mory-Chiparra WE. Parental communication and impulsivity of school adolescents during confinement for the COVID-19 pandemic. J Posit School Psychol. 2022;6(7):1602–9.

[21] Karadag M, Demir B. The impact of impulsivity and school attendance on COVID-19 spread: a web-based cross-sectional questionnaire. Psychol Sch. 2023;60(5):1581–93. 10.1002/pits.22700. 10.1002/pits.22700 PMC9088339 35572173

[22] Alper S, Bayrak F, Yilmaz O. Psychological correlates of COVID-19 conspiracy beliefs and preventive measures: evidence from Turkey. Curr Psychol. 2021;40(11):5708–17. 10.1007/s12144-020-00903-0. 32837129 10.1007/s12144-020-00903-0 PMC7324005

[23] Abdolkarimi M, Masoomi M, Lotfipur SS, Zakeri MA. The relationship between spiritual health and happiness in medical students during the COVID-19 outbreak: a survey in southeastern Iran. Front Psychol. 2022;13:974697. 10.3389/fpsyg.2022.974697. 36033099 10.3389/fpsyg.2022.974697 PMC9404234

[24] Leung CH, Mu Y. Spiritual and mental health of teenagers in Hong Kong and in Mainland China under the impact of COVID-19. Asian Educ Dev Stud. 2022;11(2):340–55. 10.1108/AEDS-04-2021-0076.

[25] Koenig HG. Maintaining health and well-being by putting faith into action during the COVID-19 pandemic. J Relig Health. 2020;59(5):2205–14. 10.1007/s10943-020-01035-2. 32409989 10.1007/s10943-020-01035-2 PMC7224109

[26] Halldorsdottir T, Thorisdottir IE, Meyers CCA, Asgeirsdottir BB, Kristjansson AL, Valdimarsdottir HB, et al. Adolescent well-being amid the COVID-19 pandemic: are girls struggling more than boys? JCPP Adv. 2021;1(2):e12027. 10.1002/jcv2.12027. 34514467 10.1002/jcv2.12027 PMC8420409

[27] Lyyra N, Thorsteinsson EB, Eriksson C, Madsen KR, Tolvanen A, Löfstedt P, et al. The association between loneliness, mental well-being, and self-esteem among adolescents in four Nordic countries. Int J Environ Res Public Health. 2021;18(14):7405. 10.3390/ijerph18147405. 34299857 10.3390/ijerph18147405 PMC8308002

[28] Lazarus RS, Folkman SC. Stress, appraisal, and coping. New York: Springer; 1984.

[29] Ahorsu DK, Adjaottor ES, Yeboah FA, Opoku Y. Mental health challenges in academia: comparison between students of the various educational levels in Ghana. J Ment Health. 2021;30(3):292–9. 10.1080/09638237.2020.1739253. 32168994 10.1080/09638237.2020.1739253

[30] Hamdani SU, Zill-E-Huma, Zafar SW, Suleman N, Um-Ul-Baneen, Waqas A, Rahman A. Effectiveness of relaxation techniques ‘as an active ingredient of psychological interventions’ to reduce distress, anxiety and depression in adolescents: a systematic review and meta-analysis. Int J Ment Health Syst. 2022;16(1):31. 10.1186/s13033-022-00541-y. 35765083 10.1186/s13033-022-00541-y PMC9238062

[31] Ingram RE, Luxton DD. Vulnerability-Stress models. In: Hankin BL, Abela JRZ, editors. Development of psychopathology: A vulnerability-stress perspective. Thousand Oaks (CA): Sage; 2005. pp. 32–46.

[32] Hörbo M, Johansson C, Garnow T, Garmy P, Einberg EL. Experiences of stress - a focus group interview study among Swedish adolescents during the COVID-19 pandemic. J Sch Nurs. 2023;39(2):189–97. 10.1177/10598405211071002. 34967254 10.1177/10598405211071002 PMC9988619

[33] Meherali S, Punjani N, Louie-Poon S, Abdul Rahim K, Das JK, Salam RA, et al. Mental health of children and adolescents amidst COVID-19 and past pandemics: a rapid systematic review. Int J Environ Res Public Health. 2021;18(7):3432. 10.3390/ijerph18073432. 33810225 10.3390/ijerph18073432 PMC8038056

[34] Nally S, Ridgers ND, Gallagher AM, Murphy MH, Salmon J, Carlin A. When you move you have fun": perceived barriers, and facilitators of physical activity from a child’s perspective. Front Sports Act Living. 2022;4:789259. 10.3389/fspor.2022.789259. 35321521 10.3389/fspor.2022.789259 PMC8937021

[35] Johansson C, Hedman Ahlström B, Barac M, Berglund T, Bador K, Kerekes N. Impact of the COVID-19 pandemic on Swedish adolescents’ mental health, psychosocial functioning, risk behaviours, and victimisation: gender differences and implications. Int J Environ Res Public Health. 2024;21(5):604. 10.3390/ijerph21050604. 38791818 10.3390/ijerph21050604 PMC11121272

[36] Chen Y, Osika W, Henriksson G, Dahlstrand J, Friberg P. Impact of COVID-19 pandemic on mental health and health behaviors in Swedish adolescents. Scand J Public Health. 2022;50(1):26–32. 10.1177/14034948211021724. 34100665 10.1177/14034948211021724 PMC8808000

[37] Kapetanovic S, Gurdal S, Ander B, Sorbring E. Reported changes in adolescent psychosocial functioning during the COVID-19 outbreak. Adolescents. 2021;1(1):10–20. 10.3390/adolescents1010002.

[38] Dumas TM, Ellis W, Litt DM. What does adolescent substance use look like during the COVID-19 pandemic? Examining changes in frequency, social contexts, and pandemic-related predictors. J Adolesc Health. 2020;67(3):354–61. 10.1016/j.jadohealth.2020.06.018. 32693983 10.1016/j.jadohealth.2020.06.018 PMC7368647

[39] Kerekes N, Bador K, Sfendla A, Belaatar M, Mzadi AE, Jovic V, et al. Changes in adolescents’ psychosocial functioning and well-being as a consequence of long-term COVID-19 restrictions. Int J Environ Res Public Health. 2021;18(16):8755. 10.3390/ijerph18168755. 34444502 10.3390/ijerph18168755 PMC8392883

[40] Pakpour AH, Eriksson M, Erixon I, Broström A, Bengtsson S, Jakobsson M, et al. The short Warwick-Edinburgh mental well-being scale (SWEMWBS) - a psychometric evaluation of adolescents in Sweden during the COVID-19 pandemic. Heliyon. 2024;10(6):e27620. 10.1016/j.heliyon.2024.e27620. 38510050 10.1016/j.heliyon.2024.e27620 PMC10950601

[41] Tennant R, Hiller L, Fishwick R, Platt S, Joseph S, Weich S, et al. The Warwick-Edinburgh mental well-being scale (WEMWBS): development and UK validation. Health Qual Life Outcomes. 2007;5:63. 10.1186/1477-7525-5-63. 18042300 10.1186/1477-7525-5-63 PMC2222612

[42] Stewart-Brown S, Tennant A, Tennant R, Platt S, Parkinson J, Weich S. Internal construct validity of the Warwick-Edinburgh mental well-being scale (WEMWBS): a Rasch analysis using data from the Scottish health education population survey. Health Qual Life Outcomes. 2009;7:15. 10.1186/1477-7525-7-15. 19228398 10.1186/1477-7525-7-15 PMC2669062

[43] Melder CA, Fischer RS, Nygren B, DeMarinis V. Validating WHOQOL-SRPB in sweden: instrument adaption for measuring existential aspects of health-related quality of life [HRQL] in secular contexts. Qual Life Res. 2016;25:100–100.

[44] Anderson JC, Gerbing DW. Structural equation modeling in practice: a review and recommended two-step approach. Psychol Bull. 1988;103(3):411. 10.1037/0033-2909.103.3.411.

[45] Dimitrov DM. Testing for factorial invariance in the context of construct validation. Meas Eval Couns Dev. 2010;43(2):121–49. 10.1177/0748175610373459.

[46] Rathakrishnan B, Singh SSB, Yahaya A, Kamaluddin MR, Aziz SFA. The relationship among spirituality, fear, and mental health on COVID-19 among adults: an exploratory research. Front Psychol. 2022;12:815332. 10.3389/fpsyg.2021.815332. 35095699 10.3389/fpsyg.2021.815332 PMC8790181

