# Supplementary material for: Impact of the COVID-19 pandemic on Swedish adolescents’ mental well-being: the role of impulsivity, sleep, spirituality, and self-esteem
Source: BMC Psychol. 2025 Dec 2;13:1350. doi: 10.1186/s40359-025-03737-2 (PMC12690890; doi:10.1186/s40359-025-03737-2)
Supplement: Supplementary file 1 — Supplementary Material 1. [file 40359_2025_3737_MOESM1_ESM.docx]

**Appendix**

**Table S1.** Invariance evaluation across sex groups on mental well-being through multigroup structural equation modelling.

| Model and comparisons | Fit statistics | | | | | | | | | |
| --- | --- | --- | --- | --- | --- | --- | --- | --- | --- | --- |
|  | χ^2^ (df) | ∆χ^2^ (∆df) | CFI | ∆CFI | TLI | ∆TLI | RMSEA | ∆RMSEA | SRMR | ∆SRMR |
| M1: Unconstrained | 10004.89 (933) * | - | 0.940 | - | 0.932 | - | 0.030 | - | 0.057 | - |
| M2: Measurement weights | 10360.32 (975) * | 355.43(42)* | 0.938 | -0.002 | 0.933 | 0.001 | 0.030 | 0 | 0.057 | 0.0004 |
| M3: Measurement intercepts | 11674.74 (1029) * | 1669.85(96)* | 0.930 | -0.01 | 0.928 | -0.006 | 0.031 | 0.001 | 0.057 | 0.0001 |
| M4: Structural weights | 11738.56 (1053)* | 1733.67(120)* | 0.929 | -0.011 | 0.929 | 0.007 | 0.030 | 0 | 0.057 | 0.0003 |
| M5: Structural Covariances | 11789.04 (1055)* | 1784.15(122)* | 0.929 | -0.011 | 0.929 | 0.007 | 0.030 | 0 | 0.057 | 0.0003 |
| M6: Structural Residuals | 11858.09 (1065)* | 1853.20(132)* | 0.929 | -0.003 | 0.929 | 0.007 | 0.030 | 0 | 0.057 | 0.0002 |
| M7: Measurement Residuals | 12965.40 (1121)* | 2960.51(188)* | 0.922 | -0.018 | 0.926 | -0.006 | 0.031 | 0.001 | 0.057 | 0.0002 |

**Note**: CFI= comparative fit index, TLI= Tucker-Lewis index, RMSEA=root mean square error of approximation, SRMR= standardized root mean square residual.

*p values are significant at P<0.05.
